# Characterization of the *Coriolopsis gallica* DyP for Its Potential to Biotransform Various Fluoroquinolones

**DOI:** 10.3390/ijms252111392

**Published:** 2024-10-23

**Authors:** Karima Staita, Imen Akrout, Julien Lambert, Annick Turbé-Doan, Anne Lomascolo, Craig B. Faulds, Héla Zouari-Mechichi, Giuliano Sciara, Tahar Mechichi, Eric Record

**Affiliations:** 1 INRAE, Aix Marseille Univ, BBF, Biodiversité et Biotechnologie Fongiques, 13288 Marseille, France; karima.staita@enis.tn (K.S.); imen.akrout@enis.tn (I.A.); julien.lambert@inrae.fr (J.L.); annick.doan@univ-amu.fr (A.T.-D.); anne.lomascolo@univ-amu.fr (A.L.); craig.faulds@univ-amu.fr (C.B.F.); giuliano.sciara@inrae.fr (G.S.); 2 Laboratoire de Biochimie et de Génie Enzymatique des Lipases, Ecole Nationale d’Ingénieurs de Sfax, Université de Sfax, 3038 Sfax, Tunisia; hela.zouari@isbs.usf.tn

**Keywords:** *Coriolopsis gallica*, dye-decolorizing peroxidase, heterologous expression, dye decolorization, biotransformation, antibiotics, fluoroquinolones

## Abstract

*Coriolopsis gallica* (*Cga*) is a white-rot fungus renowned for its ability to secrete ligninolytic enzymes that are capable of oxidizing phenolic compounds. This study aimed to investigate the biochemical characteristics of a dye-decolorizing peroxidase named *CgaDyP1* and test its ability to biotransform antibiotics. *CgaDyP1* was cloned and heterologously expressed in *Escherichia coli.* We fully characterized the biochemical properties of *CgaDyP1* and evaluated its dye-decolorizing potential to confirm that it belongs to the DyP class of enzymes. We also tested its fluoroquinolone antibiotic biotransformation potential for possible biotechnological applications. Alignment of the primary amino acid sequence with DyP homolog sequences showed that *Cga*DyP1 has high similarity with other fungal DyPs. The recombinant *Cga*DyP1 exhibited activity on substrates such as ABTS and 2,6-dimethoxyphenol (DMP) with optimal performance at a pH of 3, although activity at pH 2.5, pH 4, and pH5 diminished over time. Thermostability tests indicated that the enzyme remains stable at temperatures between 30 °C and 50 °C and retains 70% of its initial activity after 180 min at 50 °C. Tests on the effect of hydrogen peroxide on *CgaDyP1* activity found peak activity at 0.25 mM H_2_O_2_. *Cga*DyP1 decolorized five industrial dyes, and kinetics data confirmed that it belongs to the DyP class of enzymes. *Cga*DyP1 was shown to biotransform some of the 7 recalcitrant fluoroquinolone antibiotics tested here, including levofloxacin, moxifloxacin, and norfloxacin, and thus holds potential for biotechnological applications.

## 1. Introduction

The combined effects of climate change and the increasing scarcity of water resources are putting added pressure on the world’s efforts to maintain a balance between water demand and water supply while ensuring water quality. Continued human activities require a strategic approach focused on using and managing non-conventional water resources while mitigating sources of pollution. Reusing treated water is now accepted as an alternative source of water worldwide [1]. However, recent reports [2,3] have warned of the limits of current wastewater treatment systems in terms of emerging pollutants such as antibiotics. In addition, various studies have highlighted the presence of antibiotics in treated water, which has tangible consequences for ecosystems in terms of antibiotic resistance and transfer via food chains [4,5]. The rising worldwide use of antibiotics makes this issue increasingly problematic despite various plans to rationalize antibiotic use. Fluoroquinolones are a widely-used class of broad-spectrum antibiotics that are highly recalcitrant to biodegradation and frequently found to pollute freshwater and sea sediments. Environmental concentrations of fluoroquinolones are about 460 ng L^−1^ in water and 400 ng g^−1^ in sediment, which makes them the most dangerous antibiotics.

Therefore, there is an urgent need to develop sustainable methods for eliminating these pollutants. Studies have demonstrated that white-rot fungi (WRF) and their enzymes possess a high efficiency in degrading and transforming various organic pollutants. White-rot fungi (WRF), renowned as natural degraders, derive their name from their ability to degrade lignin, resulting in the characteristic white rot appearance [6]. These fungi exhibit powerful biodegradative capabilities, showing considerable potential for the removal of diverse organic pollutants [7,8]. The remarkable degradative capacity of WRF stems from their unique extracellular enzyme system, including dye-decolorizing peroxidase (EC 1.11.1.19, DyP), lignin peroxidase (EC 1.11.1.14, LiP), manganese peroxidase (EC 1.11.1.13, MnP), versatile peroxidase (EC 1.11.1.16, VP), and laccase (EC 1.10.3.2, Lac) [9,10]. Laccases have been shown to be very efficient, but they often require added chemical mediators to biotransform the most recalcitrant antibiotics, such as fluoroquinolones. There is less data available on the biotransformation of antibiotics by heme peroxidases despite their potential for various biotechnological applications [11]. However, the only two examples found in the literature illustrate DyP-driven biotransformation of β-lactams, macrolides, sulfonamides, and trimethoprim [12,13]. The heme peroxidases form a general group composed of manganese peroxidases (MnPs), lignin peroxidases (LiPs), and versatile peroxidases (VPs) that belong to the peroxidase-catalase superfamily [14], whereas dye-decolorizing peroxidases (DyPs) are part of another group, called “dimeric α + β barrel structural superfamily”, and the CDE superfamily [15]. DyPs are homodimers or heterodimers structured as a β barrel decorated with α helices, forming two ferredoxin-like motifs. Each motif contains a conserved histidine in the heme-binding site and a GXXDG signature motif, where the catalytic aspartate acts as a proton acceptor [16]. DyPs have been characterized from both bacteria [17] and filamentous fungi such as *Bjerkandera adusta* [18,19], *Pleurotus ostreatus* [20], *Auricularia auricula-judae* [21], and *Trametes versicolor* [22]. Recently, DyPs were also studied as molecular tools to explore the soil diversity of mangroves, and the main DyP identified in this environment was biochemically characterized [23]. DyPs possess a large diversity of properties and are able to oxidize phenolic compounds (2,6-dimethoxyphenol and guaiacol) and non-phenolic compounds (veratryl alcohol and Mn^2+^), together with industrial dyes such as anthraquinone (Reactive Blue 5) and azo dyes (RB5) [22,23].

We previously studied the biotransformation of the fluroquinolone levofloxacin by the white-rot fungus *Coriolopsis gallica* and used proteomic analysis to identify two groups of enzymes that are potentially involved in this process. The first group consists of 3 laccases and the second group consists of 3 DyPs [24]. Among the 3 DyP candidates, we selected the one with the highest production yield for heterologous production in *E. coli* to provide proof of concept for the use of fungal DyPs in the biotransformation of fluoroquinolones. To achieve this goal, we biochemically characterized the recombinant DyP and determined its kinetic parameters. In order to study its ability to biotransform antibiotics, 7 representatives of the fluoroquinolone class were tested under optimal conditions.

## 2. Results

### 2.1. Target Selection, Protein Production and Purification

**Figure 1:**
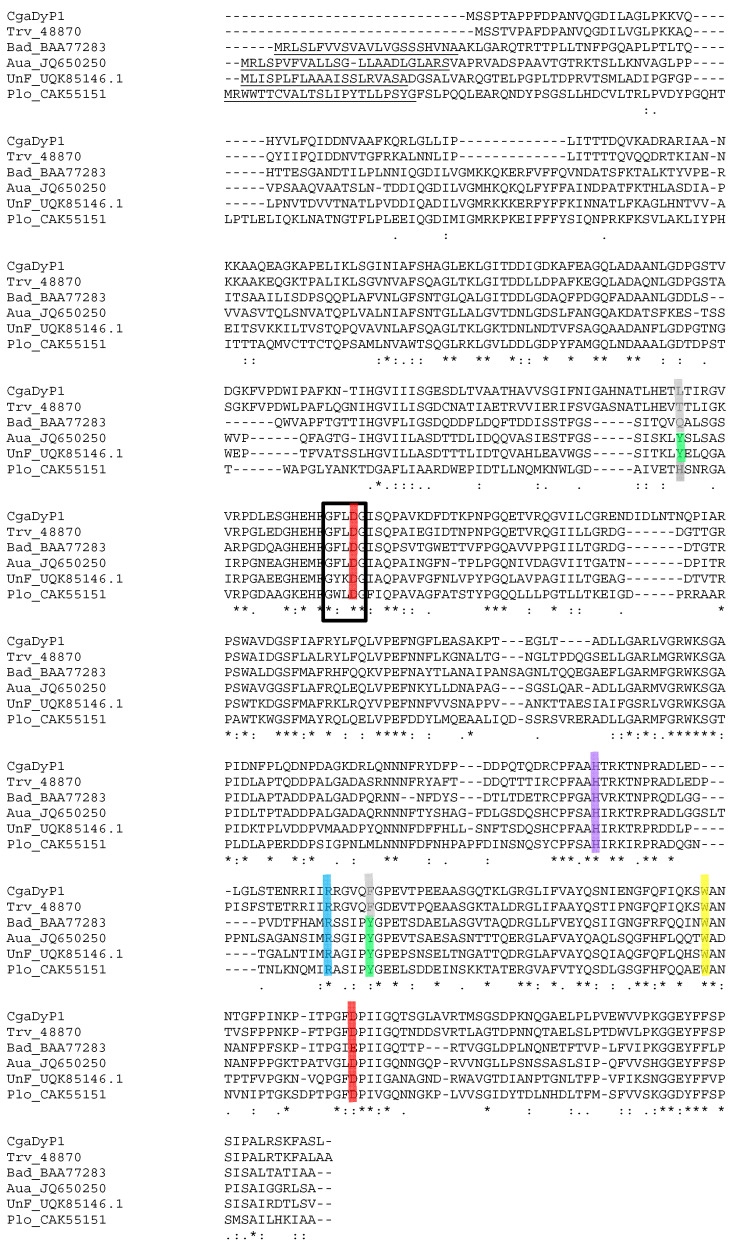
Alignment of *Coriolopsis gallica* DyP1 (*CgaDyP1*) and 5 characterized DyPs from *Trametes versicolor* (*Tve*) [22] (70.7% identity), *Bjerkandera adusta* (*Bad*) [18,19] (43.8% identity), *Auricularia auricula-judae* (*Aau*) [26] (46.0% identity), unF (uncultured fungus sampled in a mangrove) (UnF) [23] (43.4% identity), and *Pleurotus ostreatus* (*Pos*) (GenBank: CAK55151.1) (38.7% identity). Highlighted residues include (i) proximal histidine (magenta) and aspartate (red); (ii) distal-side arginine (cyan) and aspartate (red), the latter within the GXXDG signature motif (black box); and (iii) four solvent-exposed aromatic residues corresponding to two conserved tryptophans (yellow) and two tyrosines (green) sometimes substituted by other amino acids (grey). Alignment was produced with Clustal 2.1, and symbols below the sequences indicate full conservation of the same (asterisk) or equivalent residues (colon) and partial residue conservation (dot). Amino acids of the putative sequence signal are underlined.

Among the 3 DyPs identified in the *C. gallica* secretome studied for the biotransformation of levofloxacin, *Cga*DyP1 (UniProtKB number A0A2K9YND8_9APHY) was selected as the highest-produced protein. This protein has a predicted typical DyP of 52.3 kDa with an acidic pI of 5.57. SignalP prediction failed to detect any conventional signal peptide for *Cga*DyP1 as for DyPs from *T. versicolor* [22] and from the uncultured fungus sampled in a mangrove (UnF) [23]. An alignment performed with five characterized fungal DyPs (Figure 1) confirmed that *Cga*DyP1 presented the key amino acid residues characteristic of this DyP family, including (i) distal aspartate and arginine residues (Asp203 and Arg362 underlined in red and blue, respectively, in Figure 1), (ii) the proximal histidine (His342 underlined in magenta) occupying the fifth coordination position of the heme iron, and a second aspartate (Asp428 underlined in blue) [25]. Asp203, which is part of the DyP signature motif GXXDG, is necessary for enzyme activation by H_2_O_2_ [18]. We also identified surface aromatic residues putatively involved in catalysis, i.e., Trp136 and Trp411 (underlined in yellow), which are conserved in the five characterized DyPs shown in Figure 1.

### 2.2. Purification and Biochemical Characterization of CgaDyP1

The recombinant *Cga*DyP1 was purified from the *E. coli* culture medium by a two-step chromatography procedure using IMAC affinity chromatography followed by gel filtration chromatography (Table 1). *Cga*DyP1 was purified from 140 mL of a culture containing 7973.9 mg proteins, with a recovery of 2.9 mg of DyP at 88% purification yield.

**Figure 2:**
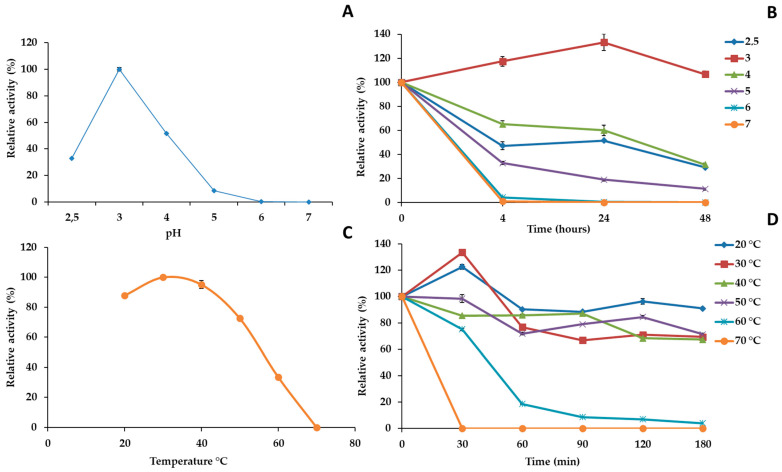
Effects of pH and temperature on *Cga*DyP1 activity and stability: (**A**) optimal pH for the oxidation of ABTS (5 mM); (**B**) pH stability in the range pH 2.5–7 for 4, 24, and 48 h incubation; (**C**) optimal temperature for the oxidation of ABTS; (**D**) temperature stability in the range 20–70 °C. Enzyme activity was measured in a 0.1 M citrate-phosphate buffer using ABTS (5 mM) as a reducing substrate and 0.25 mM H_2_O_2_ at 30 °C (and at pH 3.0 in (**C**,**D**)). Activity values were calculated as a percentage of maximum activity (set to 100%) at optimum temperature and pH. Each data point (mean ± standard deviation) is the result of triplicate experiments.

We studied the main biochemical properties of the purified *Cga*DyP1, such as the effect of temperature and pH on *Cga*DyP1 activity and its stability against the same parameters (Figure 2). *Cga*DyP1 has an acidic optimum pH of 3.0 in the range tested (Figure 2A). Its activity dropped sharply at pH 2.5 and 4 to retain just 30% and 60% of its initial activity, respectively. There was no assayable activity at higher pH in our tests. The pH stability of *CgaDyP1* was assessed by incubating the enzyme with ABTS for 4, 24, and 48 h in a pH range from 2 to 6 (Figure 2B). The enzyme proved very stable at pH 3, although its activity decreased with time at pH 2.5, pH 4, and pH 5. At pH 6 and 7, the enzyme lost all activity after 4 h of incubation.

The optimum temperature for *Cga*DyP1 activity was between 30 °C and 40 °C. However, at higher temperatures, its activity steadily decreased to 30% of initial activity at 60 °C and no activity at 70 °C (Figure 2C). The thermal stability of *CgaDyP1* was examined by testing its activity after heat-treating the enzyme at different temperatures and for various incubation times ranging from 30 to 180 min (Figure 2D). The enzyme was stable at temperatures ranging from 20 °C to 50 °C, retaining about 70% of its initial activity after 180 min of incubation at 50 °C. However, at 60 °C and at 70 °C, the enzyme lost all activity after 90 min and 30 min of incubation, respectively.

**Figure 3:**
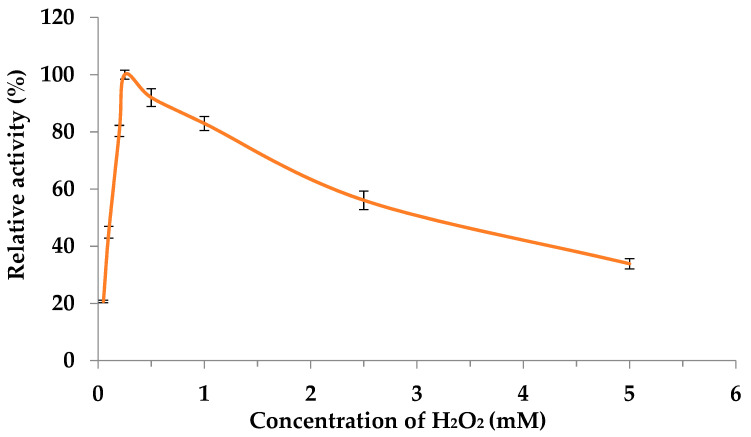
Effect of hydrogen peroxide on *Cga*DyP1 activity. Assays targeted the optimal concentration of hydrogen peroxide in standard conditions, using ABTS (5 mM) as substrate. Each data point (mean ± standard deviation) is the result of triplicate experiments.

As already described for other heme-peroxidases, DyPs are known to lose activity in the presence of H_2_O_2_ through a mechanism known as suicide inactivation [27]. The optimum concentration of H_2_O_2_ was determined by incubating the reaction mixture with concentrations of H_2_O_2_ ranging from 0.1 to 5 mM. The highest *CgaDyP1* activity was recorded at 0.25 mM H_2_O_2_ (Figure 3). Above this concentration, residual *CgaDyP1* activity decreased gradually down to 40% of its initial activity at 5.0 mM H_2_O_2_.

### 2.3. Tests on Decolorization of Industrial Dyes

The ability of *Cga*DyP1 to decolorize dye was tested against 5 industrial dyes, i.e., Acid Black (AB), Reactive Black (RB5), Disperse Blue 79 (DB79), Basic Blue 41 (BB41), and Vat Green (VG), and compared against the efficiency of the mangrove DyP, UnFDyP1, and *Tv*DyP1 (Table 2). UnFDyP1 secreted by an uncultured fungus sampled in mangrove soil and produced in *Pichia pastoris* has biochemical properties close to their terrestrial isoforms, although likely endowed with greater substrate versatility [19]. Like UnFDyP1, *Cga*DyP showed a versatile capacity to decolorize industrial dyes. *T. versicolor Tv*DyP1 heterologously produced in *Escherichia coli* was only able to decolorize AB to 75%. *Cga*DyP was able to decolorize AB with a yield of 58% but was also able to decolorize other dyes, including the recalcitrant azo dye RB5 (33%), which is carcinogenic and toxic to humans and the environment. *Cga*DyP1 was the only one of the three DyPs tested that showed some ability to decolorize VG dye (14%), a derivative of benzanthrone, a major vat dye for cotton and printing applications.

To study the substrate specificity of *Cga*DyP1, we tested 3 different substrates, i.e., the low-redox-potential dye ABTS, the phenolic aromatic compound DMP, and the recalcitrant azo anthraquinone dye RB19. *Cga*DyP1 exhibited activity against ABTS and DMP (Table 3), with *K_m_* values of 0.14 and 0.15 mM, respectively, but not against RB19. The highest catalytic efficiency (*K_cat_*/*K_m_*) was for DMP, at 26.2 s^−1^ mM^−1^, which is close to the value obtained for ABTS (21.3 s^−1^ mM^−1^).

### 2.4. Tests for Fluoroquinolone Biotransformation by CgaDyP1

**Figure 4:**
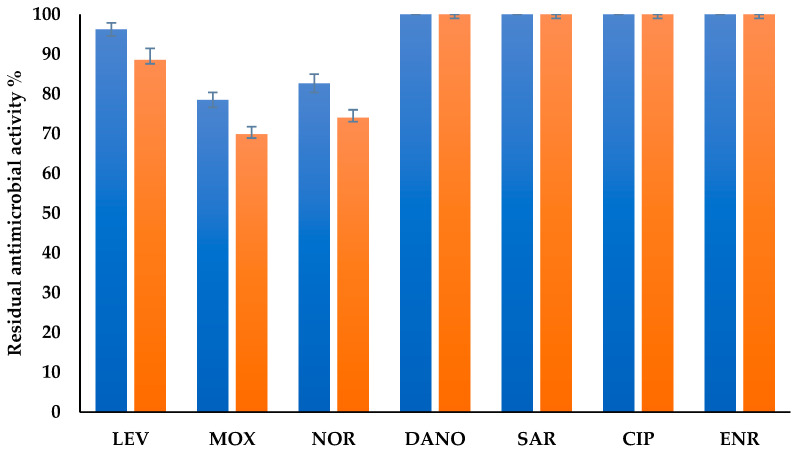
Biotransformation of fluoroquinolones by *CgaDyP1* after 24 h of incubation at 30 °C. ■ Residual antimicrobial activity after 8 h of incubation at 30 °C and at pH = 3 for 24 h; ■ Residual antimicrobial activity after 24 h of incubation at 30 °C and at pH = 3 for 24 h. LEV: levofloxacin, MOX: moxifloxacin, NOR: norfloxacin, ENR: enrofloxacin, DAN: danofloxacin, CIP: ciprofloxacin, SAR: sarafloxacin.

DyPs are well-known to oxidize industrial pollutants such as textile dyes, and more recently, tests have been performed to test whether they can biotransform antibiotics that contaminate wastewater and cause environmental problems [10]. Here we tested 7 fluoroquinolones, i.e., levofloxacin (LEV), moxifloxacin (MOX), sarafloxacin (SAR), danofloxacin (DAN), norfloxacin (NOR), enrofloxacin (ENR), and ciprofloxacin (CIP), to assess the capacity of *Cga*DyP1 to biotransform various fluoroquinolone antibiotics. The ability of *Cga*DyP1 to abolish antibiotic activity was tested against *E. coli* on a solidified spread medium. We use two control groups to assess antimicrobial activity. Control 1 consisted of the antibiotic administered without the enzyme and served as a baseline to evaluate the antimicrobial properties of the antibiotic alone. Control 2 contains the enzyme alone, without the presence of any antibiotic. As expected, this control showed 0% residual antimicrobial activity, indicating that the enzyme has no inherent antimicrobial properties when no antibiotic is applied. Together, these controls allowed us to analyze the contributions of the antibiotic and the enzyme to the overall antimicrobial efficacy observed in our experimental groups. The results presented in Figure 4 showed that CgaDyP1 was only able to biotransform three of the antibiotics, i.e., MOX, NOR, and LEV. After 8 h, the residual antimicrobial activity remained at 79% for MOX, 82% for NOR, and 96% for LEV. After 24 h, the inhibition zones continued to decrease in size, showing reductions of 69%, 74%, and 88% for MOX, NOR, and LEV, respectively. This pattern of decrease indicates that recombinant *Cga*DyP1 remained stable after 24 h of incubation and was able to effectively degrade the three antibiotics.

## 3. Discussion

*C. gallica* was previously selected for the biotransformation of levofloxacin, a third-generation broad-spectrum fluoroquinolone-class antibiotic. This fungus was shown to degrade levofloxacin by 25% in 10 days of culture, and mass spectroscopy identified the major biotransformation product as a levofloxacin N-oxide [24]. Proteomics analysis on *C. gallica* in the presence of levofloxacin identified two main groups of oxidases, including laccases and DyPs, and suggested that both enzymes were potentially involved in the biotransformation process. DyPs have primarily been used to decolorize industrial dyes but may also be valuable for biotransforming certain antibiotics [10].

Here, we selected the most-produced DyPs in the *C. gallica* secretome, called *Cga*DyP1, and used them in silico analysis to check whether they possess the main properties of a generic DyP. The primary amino acid sequence of *Cga*DyP1 aligned with sequences of biochemically characterized fungal DyPs revealed the presence of Asp203 in the conserved GXXDG-motif residues typical of all DyPs, together with Arg342, which is involved in heterolytic cleavage of H_2_O_2_ to activate the enzyme. In addition, histidine, the fifth ligand of the heme iron of DyP1, was also confirmed at position 362, with an aspartate at position 428, as for their homologs *Tve*DyP (His339 and Asp328) and *Bad*DyP (His308 and Asp391). This conserved Asp plays a key role by performing the deprotonation of H_2_O_2,_ which is crucial for compound I formation [29]. DyP1 possesses an aromatic surface composed of tryptophan and tyrosine residues to oxidize a large number of substrates. Two conserved Trp (at positions 136 and 411) were identified in the primary amino acid sequence of *Cga*DyP1. The deduced molecular weight (52.3 kDa) and the pI (5.57) calculated from the primary sequence correspond to those of typical fungal DyPs. For instance, *Tve*DyP has a calculated molecular weight of 52.2 and a pI of 5.41. Taken together, these data suggested that *Cga*DyP1 had the key features of a DyP, which prompted us to perform biochemical characterization of this potential DyP to determine its key biochemical properties and kinetic parameters and test the ability of *Cga*DyP1 to biotransform levofloxacin under appropriate conditions, in an early proof-of-concept approach. The corresponding gene was used for heterologous expression in *E. coli*, and a two-step chromatography procedure was used to purify *Cga*DyP1 with a purification yield of 88%. The purified protein was biochemically characterized by testing the effect of pH and temperature and testing its stability against the same parameters. *Cga*DyP1 has an optimum pH of 3.0 against ABTS, which is similar to the optimum pH of the mangrove DyP and the DyP from *I. lacteus* [30] and lower than the optimum pH of 3.5 for *Tve*DyP and *Pos*DyP1 [31]. *Tve*DyP and *Pos*DyP4 also showed a remarkable range of pH stability from pH 2.0 to 6.0, retaining more than 80% of initial activity for a period of 48 h. The *Cga*DyP1 protein was stable at its optimal pH, but its activity sharply decreased at higher pH, as was the case for UnFDyP, although UnFDyP was more stable at a lower pH of 2.6. The optimum temperature of *Cga*DyP1 has a broad optima temperature range of 30 °C to 40 °C, but its activity decreased to zero at 70 °C, following the same temperature curve as UnFDyP. *Cga*DyP1 was stable at temperatures ranging from 30 °C to 50 °C and retained about 70% of its initial activity after 180 min of incubation at 50 °C. In terms of enzyme inactivation, the compound tested was its own co-substrate, H_2_O_2_. Optimal activity for *Cga*DyP1 was recorded at 0.25 mM of H_2_O_2_. Residual *Cga*DyP1 activity progressively decreased with increasing concentrations, as was the case for UnFDyP and *Psa*DyP, whereas *Tv*DyP1 had an optimum H_2_O_2_ concentration of 0.125 mM [23,32]. To fully characterize *Cga*DyP1 and further confirm it is a DyP-class enzyme, we tested its ability to decolorize textile dyes on the rationale that DyPs are thought to be dye-decolorizers. The capacity of *Cga*DyP1 to decolorize industrial dyes was tested on a panel of 5 industrial dyes with different usages and chemical structures, *Cga*DyP1 was able to decolorize 58.1% of AB, 33% of RB5, 13.8% of VG, and 2.6% of DB79, thus showing a versatile ability to decolorize industrial dyes. *Cga*DyP1 was more active on AB and RB5 compared to UnFDyP, which achieved 32% decolorization on AB and 18.8% decolorization on RB5, whereas *Tv*DyP1 was only active on AB (although with 75% decolorization efficiency). *Cga*DyP1 showed higher substrate versatility and the best efficiency on RB5 (33% decolorization). The high-redox-potential dye RB5 was found to be not or only poorly oxidized by DyPs from other fungi, with the exception of *Cga*DyP1, UnFDyP, and *Aau*DyP [21]. Given the recalcitrance and high toxicity of RB5, these findings raise real prospects for practical applications of recombinant *Cga*DyP1, especially if further development, such as enzyme immobilization, enhances its real-world efficacy. However, DyPs have different decolorization effects on different types of dyes [22]. This difference is often due to the varying ability of electron-donor substituents on the aromatic ring of the dye to absorb electrons. For example, *Pos*DyP1 and TvDyP1 were unable to decolorize RB5 [22,30]. Hence, *Tv*DyP1 was also unable to degrade VG, BB, and DB. In contrast, *Cga*DyP1 was capable of decolorizing all of them except for the BB dye.

To further explore its kinetic properties, we tested *Cga*DyP1 against three well-known peroxidase substrates, i.e., ABTS, DMP, and the anthraquinone dye RB19. Recombinant *Cga*DyP1 showed good activity and affinity for ABTS and DMP but not RB19. *Cga*DyP1 had a similar affinity for ABTS to other fungal DyPs (0.12 to 0.78 mM) but lower catalytic efficiency due to lower turnover (2.95 s^−1^) than all other biochemically-characterized fungal DyPs except UnFDyP. *Cga*DyP1 performed better in the oxidation of the phenolic DMP substrate, showing equally high affinity to *Aau*DyP and similar catalytic efficiency to *Tve*DyP, although *Aau*DyP had 6.5-fold higher catalytic efficiency. *Aau*DyP also had the best catalytic efficiency on RB19 (2488 s^−1^ mM^−1^), followed by *Tve*-DyP (629.6 s^−1^ mM^−1^). *Cga*DyP1 was not able to oxidize RB19.

In conclusion, the characterized biochemical properties and ability of *Cga*DyP1 to decolorize industrial dyes confirm our hypothesis that *Cga*DyP1 from *C. gallica* is effectively a DyP. *Cga*DyP1 has the classical biochemical properties of a DyP with a large optimal temperature range and showed a very versatile ability to decolorize industrial dyes. These properties make it amenable to biotechnological applications. Its ability to efficiently decolorize many anthraquinone dyes and to degrade certain lignin substrates encouraged us to test whether it could be used to biotransform aromatic antibiotics such as fluoroquinolones, which had been demonstrated as feasible in previous work [10]. We thus tested *Cga*DyP1 on 7 fluoroquinolone antibiotics, i.e., levofloxacin, moxifloxacin, sarafloxacin, danofloxacin, norfloxacin, enrofloxacin, and ciprofloxacin, which belong to one of the most popular classes of antibiotics in the world in both human and veterinary therapy. *Cga*DyP1 was able to biotransform 79% of MOX, 82% of NOR, and 96% of LEV after 8 h of incubation at 30 °C. After 24 h, the inhibition zones continued to decrease, showing reductions of 69% for MOX, 74% for NOR, and 88% for LEV. Recent studies have demonstrated that DyPs can degrade ampicillin penicillin and other emerging pollutants [13]. However, to our knowledge, this is the first time that a DyP has been shown to degrade fluoroquinolone antibiotics. These encouraging preliminary results warrant further experiments to optimize performance by varying the enzyme concentrations and incubation times and testing out chemical and natural mediators and enzyme immobilization strategies, but the results reported here already raise prospects for the use of *Cga*DyP1 in industrial and environmental applications.

## 4. Materials and Methods

### 4.1. Cloning, Expression of DyP-Encoding cDNA, Production and Purification of Recombinant DyP

The *CgaDyP*1 sequence (GenBank number PP904445) was synthesized by Twist Bioscience (South San Francisco, CA, USA) and optimized using the codon bias in *E. coli*. The *CgaDyP*1 coding sequence was cloned into the His_SUMO_TEV vector (Addgene, Watertown, MA), and the resulting plasmid (His_SUMO_TEV-*CgaDyP*1) was used for expression in *E. coli* Lemo21 (DE3; New England Biolabs, Ipswich, MA, USA). A pre-culture was grown overnight at 37 °C in 50 mL of terrific broth (Sigma-Aldrich, Saint-Louis, MO, USA) containing 100 µg mL^−1^ kanamycin (Sigma-Aldrich) and 34 µg mL^−1^ chloramphenicol (Sigma-Aldrich). Three 1500 mL of culture medium in 4 L Erlenmeyer flasks of NZYtech medium (NZYtech, Lisbon, Portugal) used as an auto-inducing medium were then inoculated with 15 mL of overnight preculture (0.1 final OD in 1500 mL) and incubated for 24 h at 25 °C.

Cells were harvested by centrifugation at 8000 g for 10 min at 4 °C. The bacterial pellet was resuspended in 40 mL of lysis buffer (50 mM Tris, 150 mM NaCl, and 1 mM EDTA) supplemented with (Sigma-Aldrich) at 25 mg mL^−1^ lysozyme and a protease inhibitor cocktail following the manufacturer’s procedure (Roche Diagnostics, Mannheim, Germany). Cells were sonicated (Fisherbrand Model 505 Sonic Dismembrator, Fisher Scientific, Illkirch-Graffenstaden, France) at 300 Watts (60% of the maximum power) with cycles of 10 sec on and 10 sec off, repeated 6 times for a total of 2 min. Lysates were centrifuged at 8000 g for 10 min at 4 °C, and then the pH was adjusted to 7.8 before centrifuging again to remove non-soluble particles. The supernatant was then filtered with a cut-off of 0.22 μm before the purification procedure.

*Cga*DyP1 was purified using an ÄKTA Express purification system (GE Healthcare Bio-Sciences AB, Uppsala, Sweden) in two consecutive steps. First, filtered supernatant was loaded onto a 5 mL HisTrap column (GE Healthcare Bio-Sciences AB, Uppsala, Sweden) in buffer A (50 mM Tris-HCl pH 7.8, 100 mM NaCl, 10 mM imidazole) at a flow rate of 5 mL min^−1^. The HisTrap column was washed with 25 mL of buffer A, and the *Cga*DyP1 was eluted using an elution buffer B (50 mM Tris/HCl pH 7.8, 100 mM NaCl, 250 mM imidazole). The eluted proteins were dialyzed at +4 °C for 24 h using a Slide-a-laser system with 30 kDa cut-off membrane (Thermo Fisher Scientific, Waltham, MA, USA) and a dialysis buffer composed of 50 mM Tris pH 7.8 and 100 mM NaCl. The dialyzed sample was further purified by gel filtration chromatography onto a GF S75 column previously equilibrated with 50 mM buffer A and eluted with 50 mM buffer B.

Protein concentration was determined using a Nanodrop ND-2000 spectrophotometer (Thermo Fisher Scientific) by adsorption at 280 nm with theoretical molecular masses and molar extinction coefficients calculated from protein sequence using Expasy tools. A fraction of purified protein was loaded onto 10% Tris-glycine SDS-PAGE precast gel (Bio-Rad, Marnes-la-Coquette, France) to check protein purity and integrity. The molecular mass under denaturing conditions was determined using the PageRuler Prestained Protein Ladder (Thermo Fisher Scientific, Illkirch Graffenstaden, France).

### 4.2. Bioinformatics Analysis

The ProtParam tool (http://web.expasy.org/protparam/, accessed on 16 May 2024) was used to predict the theoretical pI, molecular mass, and molar extinction coefficient of DyP. Sequences were aligned with Clustal 2.1, ready for comparison (https://www.genome.jp/tools-bin/clustalw, accessed on 16 May 2024). SignalP-5.0 prediction was used to detect the presence of a signal peptide for protein secretion (https://services.healthtech.dtu.dk/services/SignalP-5.0/, accessed on 15 May 2024).

### 4.3. Activity Assays and Determination of Kinetics Parameters

Enzymatic activities were measured using a UVIKONxs spectrophotometer (Bio-TEK Instruments, Montigny-Le-Bretonneux, France) at optimal pH and at 30 °C by following the oxidation of 2,2′-azino-bis(3-ethylbenzothiazoline-6-sulfonic acid) (ABTS). Oxidation of ABTS was monitored by the generation of its cation radical (ε_420_ = 29.3 mM^−1^ cm^−1^) in 50 mM tartrate buffer at pH3 in the presence of 50 µL of DyP solution at 30 °C for 30 s. Hydrogen peroxide (0.25 mM) was added to initiate the reaction. One unit of ABTS-oxidizing activity was defined as the amount of enzyme oxidizing 1 µmoL of substrate per minute.

For the determination of kinetic parameters, ABTS and 2,6-dimethoxyphenol (DMP) (469 nm, E469 = 27,500 mM^−1^ cm^−1^) were tested in standard conditions. All enzymatic activities were measured in linear increments. The Michaelis constant, *K*_m_, together with the enzyme turnover value, *k*_cat_, were obtained by non-linear least-squares fitting of the experimental measurements to the Michaelis–Menten model. Fitting these constants to the normalized equation *v* = (*k*_cat_/*K*_m_) [*S*]/(1 + [*S*]/*K*_m_) yielded the catalytic efficiency values (*k*_cat_/*K*_m_) with their corresponding standard errors.

### 4.4. Influence of Temperature, pH, and Hydrogen Peroxide on DyP Activity and Enzyme Stability

To determine the optimal temperature, we assayed the purified DyP over the 20–70 °C temperature range in standard conditions. For the pH profiles, DyP activity was determined in 100 mM of citrate–phosphate buffer in the pH range 2.5 to 7 using ABTS as substrate at 30 °C. The effects of H_2_O_2_ on peroxidase activity were determined under standard assay conditions at the optimal pH in the range of 0.05 to 5 mM in 0.1 M citrate–phosphate buffer at 30 °C.

To define its thermal stability, aliquots of DyP were incubated at different temperatures (20–70 °C) for 30, 60, 90, 120, and 180 min. Thermal inactivation was stopped by immediately cooling the treated protein aliquot on ice, and activity was measured under standard conditions. pH stability was determined by incubating DyP in 10 mM citrate-phosphate buffer at different pH levels (2.5, 3, 4, 5, and 6) for 4, 24, and 48 h at 30 °C, and then assaying the activity in standard conditions for each substrate.

### 4.5. Decolorization Properties

The decolorization properties of *Cga*DyP1 were determined at 37 °C on 5 industrial dyes, i.e., Acid Black (AB) (560 nm; 0.005% *v/v*), RB5 (610 nm; 0.0025% *v/v*), Disperse Blue 79 (DB79) (530 nm; 0.0005% *v/v*), Basic Blue 41 (BB41) (610 nm; 0.00001% *v/v*), and Vat Green (VG) (640 nm; 0.00025% *v/v*), all supplied by SETAS (Çerkezköy, Turkey). The reaction mixture contained *Cga*DyP1 (0.125 mg mL^−1^), dye solutions (at the final concentrations described above), citrate–phosphate buffer (100 mM, pH 3), and 0.25 mM H_2_O_2_ in a total volume of 1 mL. Enzymatic dye decolorization was detected by measuring the decrease in color absorbance in 1 h. The percentage of decolorization efficiency was calculated as follows:

$$\mathrm{Decolorization}\;\mathrm{(\%)}=\frac{\left(Ai-At\right)}{Ai}\;*\;100$$

where *Ai* is the initial absorbance of the target dye, and *At* is the absorbance of the dye after each time point *t.*

### 4.6. Determination of Percentage of Antibiotic Biotransformation

Seven fluoroquinolone-class antibiotics were selected to assess their biotransformation by *Cga*DyP1. Four of these fluoroquinolones, i.e., norfloxacin, ciprofloxacin, levofloxacin, and moxifloxacin, are used in human medical therapy, and the other three, i.e., danofloxacin, enrofloxacin, and sarafloxacin, are used in veterinary medicine. All the antibiotics used in this study were purchased from Sigma Aldrich (Saint-Quentin-Fallavier, France).

The ability of *Cga*DyP1 to degrade antibiotics was tested in a 2 mL reaction mixture by incubating 25 mg L^−1^ of antibiotic with 20 U of recombinant *Cga*DyP1 in the presence of 0.25 mM H_2_O_2_ and tartrate buffer (100 mM, pH = 3). In parallel, each test was run on each antibiotic with an enzyme-free control. Samples were performed in triplicate and incubated at 30 °C for 24 h. The residual antibacterial activity of these antibiotics was evaluated by measuring their growth-inhibitory effects against *E. coli* (DH5α) [24]. Briefly, the plates containing nutrient agar were inoculated with bacterial suspension. The optical density of the suspension at 620 nm ranged from 0.08 to 0.1. Once the agar surface became dry, we made five 6-mm-diameter holes in the agar surface and spiked each hole with 50 µL of sample and control (antibiotic without *Cga*DyP1), and then incubated the plates for 24 h at 37 °C. Experiments were conducted in triplicate, and residual antibacterial activity was measured as follows:

$$\mathrm{Residual}\;\mathrm{antibacterial}\;\mathrm{activity}\;\%=\frac{\left(Dt0-Dtf\right)}{Dt0}\;*\;100$$

where Dt0 is the diameter of the inhibition zone at time 0, and Dtf is the diameter of the inhibition of the zone after 24 h of incubation.

## 5. Conclusions

In a previous study, we showed that DyPs and laccases from *C. gallica* could be involved in the biotransformation of fluoroquinolones. In this study, we carried out the production of *Cga*DyP1 to biochemically characterize the protein. We showed that *Cga*Dyp1 presented the general properties of DyPs, but we demonstrated a large versability of *Cga*DyP1 to decolorize industrial dyes, especially the recalcitrant dye RB5. Additionally, we showed that the recombinant *Cga*DyP1 was effective in biotransforming recalcitrant antibiotics of the fluoroquinolone class (moxifloxacin, norfloxacin, and levofloxacin). The presented results are of interest to valorize this class of enzymes for the bioremediation of micropollutants such as antibiotics. One possible step would be to explore enzymatic synergies between DyPs and laccases from *C. gallica* to enhance the biotransformation rate of fluoroquinolone antibiotics.

## Figures and Tables

**Table 1:** Purification of the recombinant *Cga*DyP1 produced in *Escherichia coli*. IMAC: immobilized metal affinity chromatography, GF: gel filtration chromatography.

	Volume (mL)	Total Activity (nkatal)	Protein (mg)	Specific Activity (nkatal mg^−1^)	Yield, %	Purification (Fold)
*E. coli* surprenant lysis	140	36.400	7973.9	4.6	100	1
IMAC	170	82.280	4252	19.4	226	4.24
GF	16	32.000	2.9	11.034	88	2389

**Table 2:** Percentage of decolorization of industrial dyes by the recombinant CgaDyP1 compared with the mangrove DyP UnFDyP1 [23] and the *T. versicolor* DyP *Tv*DyP [22].

Dye	*Cga*DyP1	UnFDyP1	*Tv*DyP
AB	58.1 ± 0.028	18.8 ± 0.008	75.0 ± 0.007
BB	−	−	−
RB5	33.1 ± 0.033	32.3 ± 0.009	−
DB79	2.6 ± 0.005	5.2 ± 0.005	−
VG	13.8 ± 0.008	−	−

Decolorization was determined after 1 h of incubation in citrate–phosphate buffer (100 mM, pH 3) with 0.25 mM of H_2_O_2_ at 37 °C. A ‘–’ symbol indicates no decolorization. Each data point (mean ± standard deviation) is the result of triplicate experiments.

**Table 3:** Kinetic constants of the recombinant *CgaDyP1* compared to parameters of fungal homologs against 2,2′-azino-bis(3-ethylbenzothiazoline-6-sulfonic acid) (ABTS), 2,6-dimethoxyphenol (DMP), and Reactive blue 19 dye (RB19). DyPs from *T. versicolor* [22], uncultured fungus sampled in a mangrove [23], *A. auricula-judae* [21], and *P. ostreatus* [28].

		*Cga*Dyp1	*Tve*DyP	unFDyP1	*Aau*Dyp	*Pos*Dyp1
ABTS	*K*_m_ (mM) *k*_cat_ (s^−1^) *k*_cat_/*K*_m_ (s^−1^ mM^−1^)	0.14 ± 0.02 2.95 21.3	0.29 ± 0.05 582 1989.4	0.65 ± 0.08 0.32 0.49	0.12 ± 0.01 225 1813	0.78 ± 0.07 208 267
DMP	*K*_m_ (mM) *k*_cat_ (s^−1^) *k*_cat_/*K*_m_ (s^−1^ mM^−1^)	0.15 ± 0.03 3.82 26.2	1.03 ± 0.8 87.4 85.2	0 0 0	0.70 ± 0.06 120 170.7	31.1 ± 3.8 64 2.1
RB19	*K*_m_ (mM) *k*_cat_ (s^−1^) *k*_cat_/*K*_m_ (s^−1^ mM^−1^)	0 0 0	0.04 ± 0.003 23.8 629.6	1.50 ± 0.88 3.34 2.23	0.09 ± 0.01 224 2488	0.045 ± 0.007 5 111

## Data Availability

The original contributions presented in the study are included in the article, further inquiries can be directed to the corresponding author.
